# Over-the-Scope Clip Closure of an Esophageal-Pleural Fistula Secondary to Esophageal Stent Placement: A Case Report

**DOI:** 10.7759/cureus.20696

**Published:** 2021-12-25

**Authors:** Justin Chuang, Naveena Luke, Khushbu Patel, Jordan Burlen, Ali Nawras

**Affiliations:** 1 Internal Medicine, The University of Toledo Medical Center, Toledo, USA; 2 Gastroenterology and Hepatology, The University of Toledo Medical Center, Toledo, USA

**Keywords:** esophageal cancer, over-the-scope clip, esophageal fistula, esophageal pleural fistula, otsc

## Abstract

An esophageal fistula is a pathological connection between the esophagus and another structure. The most common treatment for an esophageal fistula is airway stenting. However, several case series have demonstrated the superiority of the over-the-scope clip (OTSC) system for fistula closure. We report a case requiring multiple stent/OTSC placements in an esophageal-pleural fistula (EPF) due to underlying malignancy.

A 57-year-old male with stage IV esophageal cancer with an esophageal stent presented with three days of back pain and shortness of breath. A gastrografin was performed and showed a fistula at the proximal aspect of the pre-existing esophageal stent. A self-expandable metallic stent (SEMS) was utilized to bridge the fistula to the pre-existing esophageal stent. An esophagram two days later revealed extravasation and continuous esophageal leak. OTSC was then deployed at the fistula. A SEMS was also implanted through the patient’s pre-existing stent. Endoscopy showed persistent esophageal perforation. The initial OTSC and SEMS combination was removed. After removal, a second OTSC was placed over the fistula, allowing for complete suction of the fistula into the OTSC clip cap. We followed this by deploying another SEMS through the pre-existing stent and clipping them together. The proximal end of this new stent fully covered the fistula, creating a complete seal.

This case is notable in that successful EPF closure secondary to existing esophageal stent erosion was achieved by utilizing a properly positioned OTSC with stent-within-stent combination management.

## Introduction

An esophageal fistula is defined as the pathological connection between the esophagus and another structure [1]. The esophageal fistula occurs most often secondary to carcinoma by tumor invasion through the wall of the esophagus and a neighboring structure but can also occur secondary to airway stent placement [1]. Esophageal fistulas can at times be inoperable and may precipitate malnutrition and infection [2]. Therefore, determining the best method to treat this condition is imperative for improved quality of life. Currently, the most common treatment for esophageal fistula is esophageal stenting [1-2]. However, several case series have demonstrated the superiority of the over-the-scope clip (OTSC) system for fistula closure [2-3]. Its ergonomic shape has allowed the OTSC system to allow safe passage of food while swallowing and concomitantly surpasses conventional endoclips in closure power [2-3]. Further case studies have described primarily successful closure via OTSC in gastrointestinal (GI) lesions with notably few complications [4]. We report an interesting case for OTSC closure of an esophageal-pleural fistula (EPF) secondary to esophageal stent placement.

This article was previously presented as an abstract poster presentation at The American College of Gastroenterology, October 2021, Volume 116, Issue p S1047.

## Case presentation

Our patient was a 57-year-old male with a significant past medical history for stage IV esophageal cancer and a previously placed esophageal stent (partially covered esophageal self-expandable metallic stent 1.8 cm x 12.3 cm) one year prior to presentation. He presented with a three-day history of back pain, right-sided chest pain, and shortness of breath. A computed tomography (CT) scan revealed a right-sided pleural effusion and pneumothorax requiring two thoracostomy tube placements.

**Figure 1:**
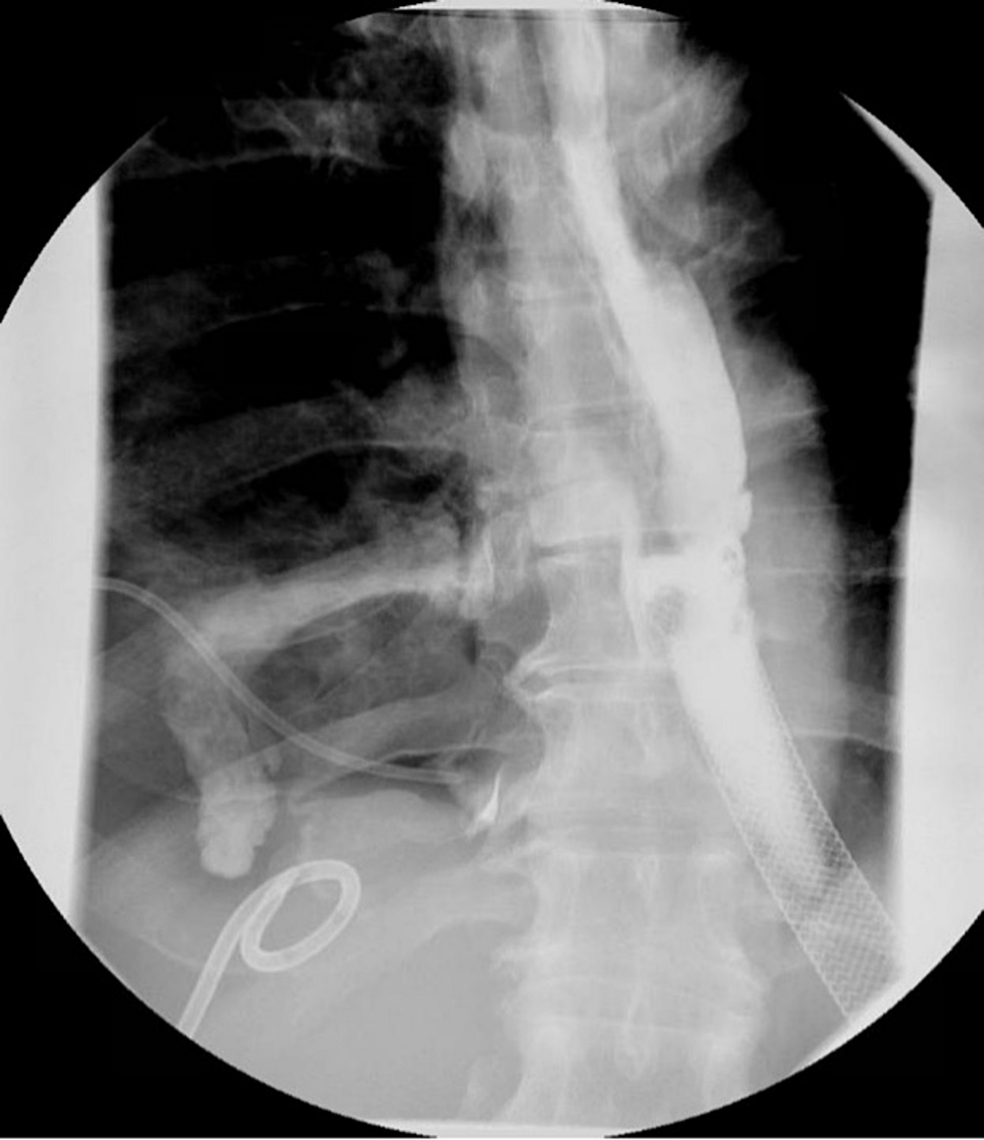
Upper GI series with gastrografin showing contrast extravasation due to EPF GI: gastrointestinal

At the time of transfer, the patient was tachycardic, tachypneic, requiring 4L oxygen via nasal cannula, and admitted to the surgical intensive care unit. Physical examination was unremarkable for any acute changes. Due to the drainage of green bilious output from the chest tube, an upper gastrointestinal series with gastrografin was performed. This demonstrated a fistula at the proximal aspect of the pre-existing esophageal stent likely caused by the stent edge eroding into the wall of the esophagus (Figure 1).

**Figure 2:**
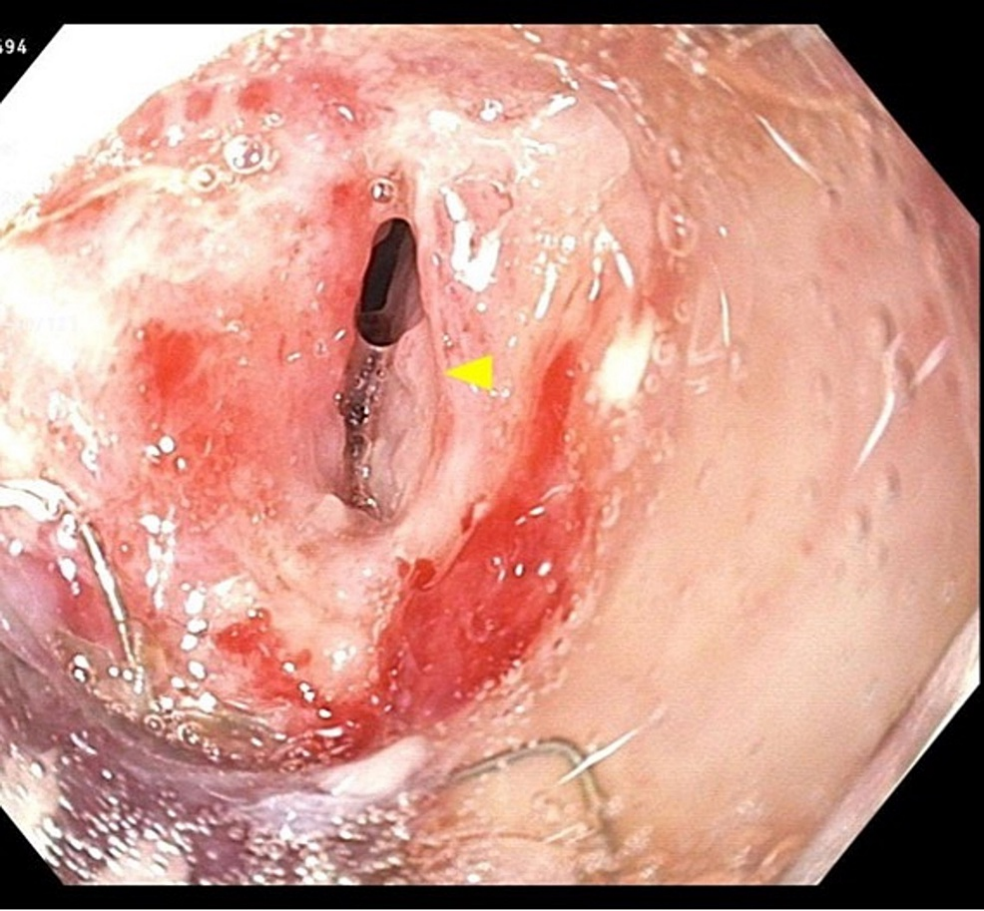
Endoscopic images confirming an esophageal fistula adjacent to the proximal end of the esophageal stent

Due to concerns for esophageal perforation, a decision was made to perform an endoscopic stent within stent placement. Introduction of the endoscope into the esophagus demonstrated a fistula at the middle third of the esophagus, 38 cm from the incisors. At this point, a fully covered 1.8 cm by 12.3 cm self-expandable metal stent (SEMS) was utilized to bridge the fistula to the pre-existing esophageal partial stent and secured with three endoclips. Unfortunately, a repeat esophagram two days later revealed barium extravasation and continuous esophageal leak along the superior margin of the newly placed stent into the right pleural space. The OTSC clip was then deployed at the fistula via esophagogastroduodenoscopy (EGD). With the fistula located posterior to the original stent, grasp of the fistula into the cap of the bear claw allowed for only partial closure. CT scan showed persistent esophageal perforation and contrast extravasation from the fistula. Endoscopic images confirmed an esophageal fistula adjacent to the proximal end of the esophageal stent (Figure 2).

**Figure 3:**
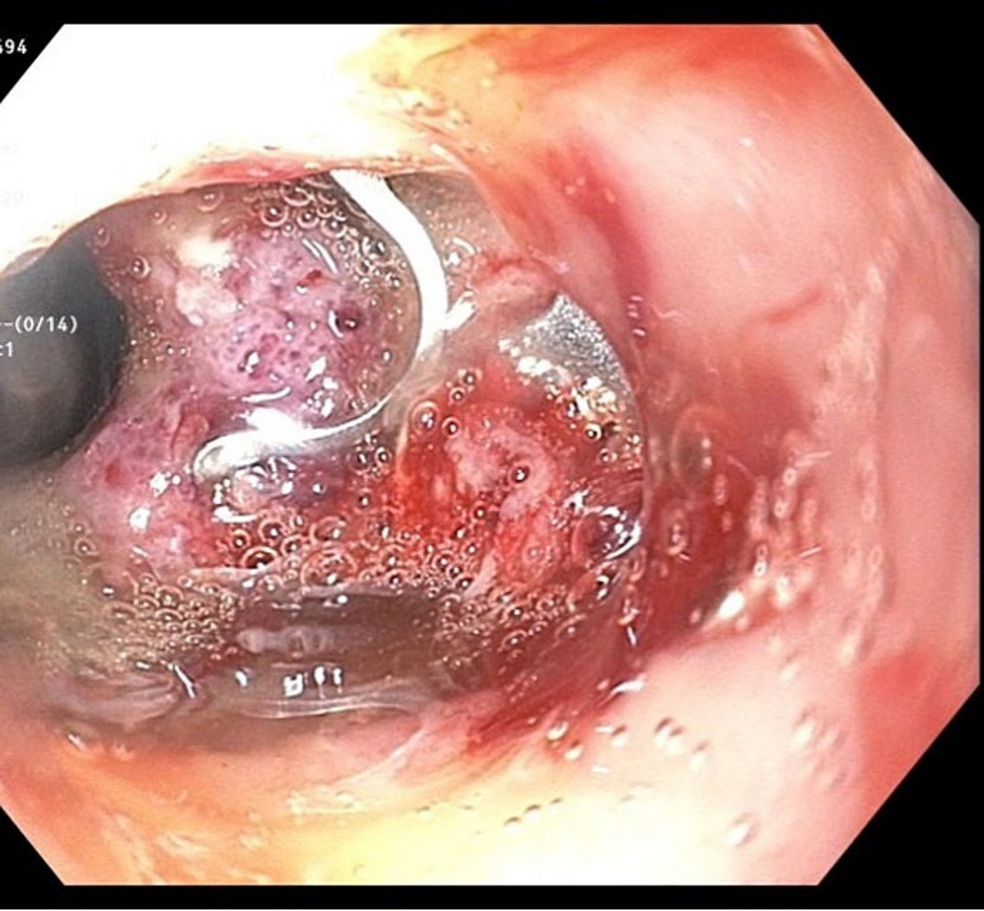
Placement of OTSC clip over fistula allowing for complete closure of the fistula OTSC: over-the-scope clip

**Figure 4:**
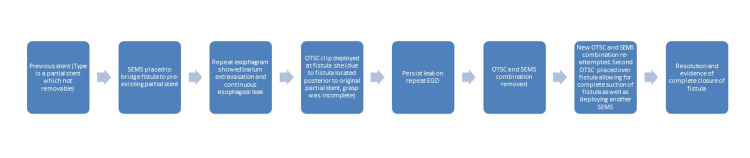
Flow diagram for the sequence of events

With the ongoing deterioration of the patient’s health, the initial OTSC and SEMS combination was removed. After removal, a second OTSC was placed over the fistula allowing for complete suction of the fistula into the OTSC clip cap (Figure 3). We followed this by deploying another fully covered 2.3 cm by 15.5 cm SEMS through the pre-existing stent and clipped them together. The proximal end of this new stent fully covered the fistula, creating a complete seal. A follow-up CT scan with oral contrast demonstrated an improved appearance of the pleural fluid and gas with no signs of drainage from the site of the previous fistula. Figure 4 shows the sequence of events.

## Discussion

The standard of care for management is dependent upon the etiology of the esophageal fistula. Surgical repair in malignant esophageal fistula is contraindicated due to the debilitated condition of patients [5]. The current frequently utilized palliative practice consists of endoscopic placement of a SEMS within the esophagus [2]. With stent placement, evaluation of potential complications is essential. As seen in our patient, the formation of an EPF is a major concern.

The OTSC technique is appealing for its speed, accessibility with insertion, and lasting closure of fistulas [4]. The OTSC system, first introduced by Kirschniak and colleagues, is a bear claw-shaped nitinol clip that is now being used for the successful closure of fistulas, leaks, and perforations [2]. This system includes the clip attached to the tip of the endoscope, which is then deployed over the orifice of the lesion allowing the “claws” of the clip to grasp the mucosa firmly and to seal the fistula shut [2].

For our patient, the original management with the stent-within-stent placement was ineffective in providing resolution. This case is notable in that successful EPF closure secondary to existing esophageal stent erosion was achieved by utilizing a properly positioned OTSC with stent-within-stent combination management.

A study by Von Renteln et al. discussed OTSC system failure in initial closure attempts of one esophageal pulmonary fistula and one jejunal cutaneous fistula due to fibrosis and scarring at the fistula site [6]. They demonstrated that a persistent fistula may be related to the condition of the mucosal tissue depending on whether it is post-malignancy, chemotherapy-induced, or from iatrogenic trauma. Understanding the limitations of closure modalities regarding location and tissue condition will be important to consider in order to prevent unsuccessful interventions. This relatively newly developed OTSC technique, although with promising results, requires further research to determine its efficacy in the long-term closure of EPF. Listed below are other documented EPF closures by OTSC found in the literature (Table 1).

**Table 1:** Documented EPF closures by OTSC EPF: esophageal-pleural fistula; OTSC: over-the-scope clip

Study	Type	Etiology for the cause of EPF	Number of attempts with OTSC	Complications	Successful OTSC placement
Kim et al. [7]	Case report	Traffic accident	1	None	Yes
Khamaysi et al. [8]	Case report	Variceal sclerotherapy	2	Initially recurrent pleural drainage	Yes
Correia et al. [9]	Retrospective study	Mallory-Weiss tear	1	None	Yes
Beoletto et al. [10]	Case report	Pneumonectomy	1	None	Yes
Zhang et al. [11]	Case report	Pneumonia	1	None	Yes

## Conclusions

Treatment for an esophageal fistula is imperative, as unmanaged fistulas are associated with high morbidity and mortality secondary to life-threatening complications, including sepsis, lung abscess, or acute respiratory distress syndrome. This presentation of multiple stent placements and multiple OTSC placements in EPF due to malignancy is unique. A thorough search was conducted on PubMed, Cochrane Library, and Google. Keywords included “esophago-pleural fistula, over-the-scope clip system, self-expandable metallic stent” and no similar cases were found. Hence, we hope it may provide useful information for future gastroenterologists who may one day face a similar patient and build upon their knowledge base for treatment options.
